# Omics Investigations of Prostate Cancer Cells Exposed to Simulated Microgravity Conditions

**DOI:** 10.3390/biom15020303

**Published:** 2025-02-18

**Authors:** Herbert Schulz, Fatima Abdelfattah, Anna Heinrich, Daniela Melnik, Viviann Sandt, Marcus Krüger, Markus Wehland, Per Hoffmann, José Luis Cortés-Sánchez, Matthias Evert, Katja Evert, Daniela Grimm

**Affiliations:** 1 Department of Microgravity and Translational Regenerative Medicine, Otto von Guericke University, 39106 Magdeburg, Germanymarcus.krueger@med.ovgu.de (M.K.);; 2 Research Group “Magdeburger Arbeitsgemeinschaft für Forschung unter Raumfahrt- und Schwerelosigkeitsbedingungen” (MARS), Otto von Guericke University, 39106 Magdeburg, Germany; jose.cortes@med.ovgu.de; 3 Institute of Human Genetics, University Hospital of Bonn, 53127 Bonn, Germany; 4 Human Genomics Research Group, Department of Biomedicine, University Hospital Basel, 4031 Basel, Switzerland; 5 Institute of Anatomy, Medical Faculty, Otto-von-Guericke-University Magdeburg, Leipziger Straße 44, 39120 Magdeburg, Germany; 6 Institute for Pathology, University Regensburg, 93053 Regensburg, Germany; 7 Department of Biomedicine, Aarhus University, 8000 Aarhus, Denmark

**Keywords:** omics, gene expression, DNA methylation, simulated microgravity, prostate cancer, cell culture, fibronectin, random positioning machine

## Abstract

Prostate cancer (PC) is the most diagnosed cancer in males across the globe. Following the formation of metastasis, PC is linked to a notable decline in both prognosis and survival rates. Three-dimensional multicellular spheroids (MCSs) of a prostate adenocarcinoma cell line were generated in a three-day simulated microgravity environment (s-µ*g*) to serve as a model for metastasis and to derive transcriptional and epigenetic PC candidates from molecular biological changes. With an FDR of 10^−3^, we detected the most differentially expressed genes in the two comparisons’ adherent cells (AD) to MCSs (N = 751 genes) and 1*g* control cells to MCSs (N = 662 genes). In these two comparisons, genes related to cell cycle, angiogenesis, cell adhesion, and extracellular space were consistently found to be significantly enriched in GO annotations. Furthermore, at a 5% FDR significance level, we were able to identify 11,090 genome-wide differentially methylated positions (DMPs) and one differentially methylated region in the *SRMS* gene in the 1*g* vs. AD comparison, as well as an additional 10,797 DMPs in the 1*g* vs. MCSs comparison. Finally, we identified five s-µ*g*-related positive enrichments of transcription factor binding sites for AR, IRF1, IRF2, STAT1, STAT2, and FOXJ3 close to the DMPs.

## 1. Introduction

Prostate cancer (PC), the most common cancer in men worldwide, is divided into adenocarcinomas, the rare squamous cell carcinoma, and stromal tumors and is usually followed by metastases to the liver, lung, and bone [1]. Unfortunately, the formation of metastases, particularly in PC, is associated with a significant deterioration in prognosis and survival [2]. For 2024, the American Cancer Society estimated about 299,010 new PC cases and 35,250 deaths from this cancer type in the US [3]. Therefore, it is necessary to investigate the functional causes of the disease, which was the objective of this study and was performed by means of multi-omics analyses and modern microgravity (µ*g*) technology.

Today, cancer research in µ*g* is a hot topic. The international space station (ISS) provides a unique µ*g* environment for cancer research. Researchers try to increase their understanding of the disease, to identify potential biomarkers for early cancer diagnosis, and to find novel treatments for various tumor types [4,5,6].

It has been known for a long time that cancer cells exposed to real µ*g* (r-µ*g*) in space or simulated µ*g* (s-µ*g*), created by so-called ground-based facilities (GBF) acknowledged by NASA, ESA, and other space agencies, exhibit numerous cellular changes [6,7]. Examples of µ*g* simulators are the NASA-developed Rotating Wall Vessel, the two-dimensional (2D) and three-dimensional (3D) clinostat, and the random positioning machine (RPM) described earlier in detail [6]. The RPM is a device that rotates biological samples/cell cultures (flasks, slides, hardware container) along two independent axes to change their orientation in space in complex ways and thus eliminate the effect of gravity. A stay in orbit induces 3D growth of FTC-133 follicular thyroid cancer cells and the formation of 3D multicellular spheroids (MCSs) and impacts cell signaling, gene expression, and protein synthesis in several biological processes [7,8]. A similar result was obtained for PC-3 cells exposed to an RPM for short periods, 24 h, 3 days, as well as 5 days [9,10]. PC-3 cells formed spheroids when cultured under s-μ*g* conditions [10]. These MCSs engineered in μ*g* represent an interesting novel model to study the early phases of metastasis in vitro [6]. The engineered in vitro tumor spheroids show a high potential for preclinical drug targeting and cancer drug development. It is possible to examine the processes of cancer progression and metastasis on a molecular level.

The µ*g* environment enables researchers to study mechanisms and pathways controlling the growth and function of cancer cells in ways that are not possible on Earth.

**Figure 1:**
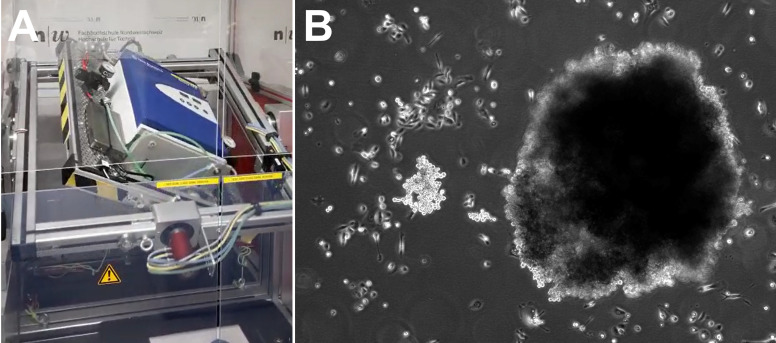
Simulated microgravity for the generation of 3D spheroids. (**A**) The iRPM. An incubator keeping cell culture flasks at optimal culture conditions is rotated by two independent cardanic frames, ensuring an efficient randomization of the gravity vector for the samples over time. (**B**) Phase contrast picture of the resulting 3D spheroids after 3 d on the iRPM.

As in our previous PC studies [9,10], we used random positioning machine (RPM)-engineered (Figure 1A) three-dimensional (3D) multicellular spheroids (MCSs, Figure 1B) as a model for metastasis to investigate molecular biological changes within the cell composition. Already in the first studies of prostate cancer cells in s-µ*g*, these were described as less aggressive and proliferative, slower growing, and more highly differentiated [11], which is why µ*g* represents an interesting study condition for PC cells. PC-3 PC cells were subjected to a three-day exposure to s-µ*g* using an RPM (Figure 1A). The generated 3D MCSs were compared to two-dimensionally (2D) growing adherent cells regarding gene expression (RNAseq) and DNA methylation (EPIC bisulfite methylation array). The resulting transcriptional and epigenetic alterations were then in silico associated with transcription factor binding site (TFBS) compounds and finally with known PC implications and previous µ*g* findings.

In contrast to 2D monolayers, 3D MCSs are structures exhibiting several characteristics observed in vivo, including architectural complexity, cell–cell interactions, oxygen and nutrient transport, and conditions of tumorigenesis. Three-dimensional spheroid formation promoted proliferation, migration, and tube formation of endothelial cells and has been tested in burn injury mice for mesenchymal stem cell (MSC)-based wound healing therapy [12]. Ovarian cancer MCSs showed a proliferation-stagnant but invasive phenotype exhibiting an upregulated Bcl-2, which contributes to cisplatin resistance [13]. It should be noted that various other techniques can be used to generate MCSs. In oral squamous cell carcinoma (OSCC) cell lines, the liquid overlay technique has been successfully used to generate MCSs [14]. In OSCC, MCSs have been described to mimic in vivo tumors with proliferative cells in the periphery and hypoxic centers, where cells under hypoxic conditions show a 3-fold decrease in fibronectin (FN1) protein expression [15]. MCSs of cancer cells often acquire multi-drug resistance and serve therefore as an ideal experimental model for drug treatment. For instance, MCSs deriving from glioma cell lines and from primary cultures of glioblastoma multiforme tumors exhibit a resistance to tamoxifen (TAM)-induced cytotoxicity [16]. Furthermore, the hypoxia status in dense MCSs in the breast cancer cell line MCF-7 has been identified as a potential contributing factor to their drug resistance [17].

The translational implication of s-µ*g* exposure of human cells is its impact on biological processes, and particularly the altered growth behavior of cancer cells. Within 24 h, one part of the cells will aggregate and form 3D tumor spheroids, whereas the other one remains growing adherently on the bottom of the culture flask in RPM samples [7,9,10,18]. These MCSs can be used as metastasis models for different cancer types [6]. Use of MCS models is known for pharmacological or toxicological studies, radiation experiments, or application as co-cultures to study angiogenesis or metastasis. Omics investigations with a focus on mechanisms can improve the current knowledge about cancer progression, epithelial to mesenchymal transition, and metastasis.

The integration of data from multiple omics levels represents a promising approach to elucidating the underlying biological mechanisms and identifying potential key factors. At present, the number of multi-omics studies, and particularly those related to epigenomics, conducted in relation to μ*g* and cancer is relatively limited. However, the open-access NASA GeneLab provides multi-omics analysis capabilities related to spaceflight response [19]. Reanalysis of GeneLab mouse RNAseq datasets demonstrated that spaceflight induces an asynchronous expression of clock genes across tissues [20], while disturbances in circadian rhythm have been identified as a risk factor for cancer in humans [21]. The analysis of cell-free DNA (cfDNA), cell-free mitochondrial DNA (cf-mtDNA), and exosomes in a one-year International Space Station (ISS) mission of a fifty-year-old astronaut and, as a control, his identical twin brother on Earth revealed no differences in cfDNA concentration. cfDNA serves as an indicator of cancer. Three years after his return from the ISS, Bezdan et al. identified an association between exosomes and the stress and cancer risk-related 20S proteasome [22]. Chromatin immunoprecipitation DNA sequencing (ChIP-Seq) of primary human M1 macrophages revealed a rapid downregulation of H3K4me3 binding to immunoregulatory genes under the gravitational stress of a parabolic flight (PF) [23]. Interestingly, we found a significant enrichment of differentially expressed genes of immune response (GO:0006955) and inflammatory response (GO:0006954) in PC-3 cells under PF conditions [4].

The aim of this study is to profile the prostate tumor cell line PC-3 for aberrations in either RNA or epigenetic DNA methylation states accompanying 3D MCS aggregation status or s-µ*g* sensitivity to provide candidate genes and gene interactions as potential PC biomarkers.

## 2. Materials and Methods

### 2.1. Cell Culturing

In 1979, PC-3 cells (ECACC 90112714) were isolated from a bone metastasis from a 62-year-old male Caucasian patient suffering from grade IV prostate adenocarcinoma [24,25,26]. The cells were purchased from the European Collection of Authenticated Cell Cultures (ECACC, Salisbury, UK).

RPMI 1640 medium (Thermo Fisher Scientific, Darmstadt, Germany), supplemented with 10% fetal calf serum (FCS) (Sigma Aldrich, Steinheim, Germany) and 1% penicillin-streptomycin (Life Technologies, New York, NY, USA) was used for cell cultivation. The cells were cultured in T75 cm^2^ flasks (Sarstedt, Nümbrecht, Germany) at 37 °C and 5% CO_2_, and the medium was changed every two to three days.

### 2.2. Simulated Microgravity on the iRPM and Sample Collection

For the experiments in the incubator RPM (iRPM, Fachhoschule Nordwestschweiz, Windisch, Switzerland, Figure 1A), a total of 24 T25 cm^2^ flasks with vented caps (Sarstedt, Nümbrecht, Germany) were each seeded with 2·10⁶ cells and filled with RPMI 1640 medium (Thermo Fisher Scientific, Darmstadt, Germany), supplemented with 10% fetal calf serum (Sigma Aldrich, Steinheim, Germany) and 1% penicillin-streptomycin (Life Technologies, New York, NY, USA). The flasks were maintained at 37 °C, 5% CO₂ for 24 h to promote cell adhesion.

Afterwards, the flasks were fully filled with growth medium, carefully avoiding air bubble formation, and the bottle caps were securely sealed with parafilm to prevent leakage during rotation in the RPM. Six flasks for each group (RNA sequencing and DNA methylation) were placed into the iRPM. The iRPM was then operated in real random mode, with a mean angular velocity of 60°/s, resulting in a simulated gravity value of 0.01 *g*. In contrast, two additional sets of six flasks were maintained under standard 1 *g* conditions at 37 °C with 5% CO₂ as static controls. After three days, the cell culture supernatants were collected into 50 mL tubes and centrifuged at 4 °C to isolate the multicellular spheroids (MCSs). The MCSs were then washed with phosphate-buffered saline (PBS) and centrifuged again. Then, 2 mL of RNA*later* Stabilization Solution (Thermo Fisher Scientific, Darmstadt, Germany) was added to the MCS pellets intended for RNA isolation, while 2 mL of PBS was added to the MCS pellets for DNA isolation. Adherent cells were mechanically detached using 10 mL of ice-cold PBS and cell scrapers. The resulting cell suspensions were collected in 15 mL tubes and centrifuged, and the supernatant was discarded. Finally, either 2 mL of the RNA*later* Stabilization Solution or PBS was added to the samples, which were then stored at 4 °C until further processing for RNA or DNA isolation, respectively.

### 2.3. RNA Isolation

The suspensions of MCSs and cells in RNA*later* were diluted 1:5 with PBS to facilitate sedimentation during centrifugation. Then, the supernatant was removed via centrifugation. RNA isolation was performed using the RNeasy Mini Kit (Qiagen, Hilden, Germany) following the manufacturer’s instructions. Briefly, 600 µL of an RLT lysis buffer was added to the pellets, and the cells or MCSs were lysed first by pipetting up and down and then passing the lysate through a 20-gauge needle on a syringe until the mixture was homogenous to the eye. Subsequently, 600 µL of 70% ethanol was added, and 700 µL of this mixture was transferred to the RNeasy Mini spin column, which was centrifuged for 15 s at 8000× *g*. This was repeated until the complete lysate was passed through the column. Afterward, 700 µL of RW1 buffer and 2 × 500 µL of RPE buffer were added, with a centrifugation step between each buffer. To elute the RNA, 35 µL of RNase-free water was pipetted directly onto the spin column membrane, and the column was then transferred to a new Eppendorf tube. Finally, a last centrifugation (1 min at ≥15,000× *g*) was performed to detach the RNA from the membrane. The quality and concentration of RNA in each sample was evaluated using a NanoPhotometer™ N60 (Implen, Munich, Germany).

### 2.4. RNA Sequencing

After quality control, the extracted RNA was converted into libraries using the TruSeq™ RNA Library Prep Kit v2 (Illumina, San Diego, CA, USA) following established protocols. These libraries were sequenced on a NovaSeq 6000 (Illumina, Berlin, Germany) platform, generating over 20 million paired-end 100 bp reads. The quality of the sequencing data was evaluated based on reading counts and quality scores. Unaligned reads were then aligned to the hg38 genome using STAR 2.7.11a [27] and ENSEMBL version 99 [28], allowing for a maximum of ten mismatches and up to ten different positions while excluding secondary alignments.

### 2.5. Gene Expression Analysis

To capture DEGs, we used the Bioconductor version 3.17 R package DESeq2 version 1.40.2 [29]. DESeq2 uses the uniquely mapped RNAseq counts to an exon based on a negative binomial generalized linear model (GLM). Principal component analysis (PCA) was employed to ascertain batch effects and the sample expression distribution using the plotPCA function of DESeq2. The principal axis transformation of PCA serves to simplify the gene expression matrix to a smaller number of most meaningful linear combinations (the principal components) using the eigenvectors of the covariance matrix. The R package DynaVenn (https://ccb-compute.cs.uni-saarland.de/dynavenn, accessed on 13 February 2025) [30] was utilized to ascertain the most significant overlap between the 1*g* to AD and the 1*g* to MCS comparison. The DEG sets were previously ordered by *p*-value. For integrated post hoc analysis of enrichments and networks of differentially expressed genes, we employed the Database for Annotation, Visualization and Integrated Discovery (DAVID Knowledgebase v2024q2) [31] and the protein-to-protein interaction (PPI) database STRING v. 12.0 [32], respectively.

### 2.6. DNA Isolation

DNA was extracted from three distinct groups: MCS, adherent cells from RPM cultures, and adherent cells from the 1*g* control group. The extraction utilized the DNeasy^®^ Blood and Tissue Kit (Qiagen, Hilden, Germany). Initially, cell pellets from the three groups were centrifuged at 300× *g* for 5 min and then resuspended in 200 µL of PBS. Following the manufacturer’s instructions, the samples were lysed with 20 µL of proteinase K. After lysis, 200 µL of buffer AL was added, and the samples were incubated at 56 °C for 10 min. Post-incubation, 200 µL of 96–100% ethanol was added to the mixture, which was then mixed thoroughly by vortexing.

The resulting homogeneous solution was transferred to a DNeasy Mini (Qiagen, Hilden, Germany) spin column and centrifuged at ≥6000× *g* for 1 min. The flow-through was discarded, and each spin column was placed into a new 2 mL collection tube. Next, 500 µL of Buffer AW1 was added, and the samples were centrifuged again at the same speed for 1 min. The flow-through was discarded once more, and the spin columns were transferred to new 2 mL collection tubes. Subsequently, 500 µL of Buffer AW2 was added, and the samples underwent centrifugation for 3 min at 20,000× *g*. Finally, the spin columns were moved to new 1.5 mL microcentrifuge tubes, and 200 µL of Buffer AE was added. After 1 min incubation at room temperature, the DNA was eluted by centrifugation at ≥6000× *g* for 1 min. The quality and quantity of the purified DNA were evaluated, ensuring an A260/A280 ratio of ≥1.8.

### 2.7. Preparation and Filtering of DNA Methylation Data and DMP and DMR Analyses

Bisulfite conversion of genomic DNA was performed using the Zymo EZ DNA Methylation Kit (kit #D5001; Zymo Research Corp., Irvine, CA, USA) adhering to the manufacturer’s instructions. A total of 500 ng of bisulfite-converted DNA was analyzed using the Illumina Infinium EPIC BeadChip (Illumina, San Diego, CA, USA) following the provided guidelines. Signal intensities from the resulting images were extracted using GenomeStudio and the EPIC manifest (Illumina, San Diego, CA, USA).

The EPIC CpG methylation data underwent filtering, quality control, and normalization using the ChAMP version 2.30.0 R library [33]. Furthermore, additional filtering of cross-reactive probes was conducted using the R library maxprobes, which is included in the package’s “devtools”. The probes were filtered in detail as follows: probe detection *p*-value > 0.01 (-5047 probes), probes with a beadcount <3 in at least 5% of samples (–39,808 probes), filtering non-CG probes (–2785 probes), SNP containing CG probes (–94,017 probes), crossreactive probes (ChAMP –11 probes), additional cross-reactive probes by maxprobes (–3117 probes). After quality filtering, 721,133 CpGs were included in the differential methylation position (DMP) and differential methylation region (DMR) analyses using the functions champ.DMP and champ.DMR (bumphunter) included in ChAMP.

### 2.8. DNA Methylation Enrichment Analyses

Enrichment analyses were performed using the GOmeth function of the missMethyl version 1.34.0 Bioconductor R package [34]. TFBS enrichment analyses were performed using chi-square statistics of DMP vs. non-DMP CpGs over the Probes.motif.hg19.EPIC dataset of the ELMER.data bioconductor package. Probes.motif.hg19.EPIC was generated using HOMER with a *p*-value < 10^−4^ to scan a ± 250 bp region around each probe using the HOCOMOCO [35] collection of position weight matrices.

### 2.9. Histology

Spheroids were collected in centrifugation tubes (Sarstedt), centrifuged at 125× *g* for 5 min at 4 °C, and fixed with 4% paraformaldehyde (PFA). All samples were stored at 4 °C. Spheroids were embedded in paraffin and sectioned with a microtome into 3 µm sections.

### 2.10. Immunohistochemical Staining, Microscopy and Analysis

The samples were first deparaffinized and rehydrated before unmasking was performed for 30 min at 99 °C. Samples were then permeabilized in 0.3% Triton X-100 (Carl Roth, Karlsruhe, Germany) in 0.1 M phosphate buffer (PB) for 30 min and blocked in 3% bovine serum albumin (BSA, Carl Roth, Karlsruhe, Germany) in 0.1 M PB with 1:10 normal goat serum (NGS, Agilent, Santa Clara, CA, USA) for 1 h to prevent non-specific binding. Slides were labeled overnight at 4 °C with the primary antibodies (Table S1) diluted in 0.1 M PB with 1:10 NGS. The next day, the sections were washed three times with 0.1 M PB, incubated with the secondary antibodies (Table S1) for 1 h at room temperature, and then washed again three times with 0.1 M PB. For nuclear DNA staining, samples were incubated with 100 ng/mL DAPI (4′,6-diamidino-2-phenylindole; Thermo Fisher Scientific) in 0.1 M PB for 10 min at room temperature. Finally, the slides were washed three times with 0.1 M PB and mounted with Fluoromount^TM^ (Sigma-Aldrich, St. Louis, MO, USA).

After staining, the slides were analyzed in a ZEISS LSM 800 confocal laser scanning microscope (Carl Zeiss, Jena, Germany) using the ZEISS Airyscan detector mode and ZEN 3.4 software (Carl Zeiss). The laser intensity was optimized for each antibody–wavelength combination. The images were processed, and the same setting was used for all files to allow comparison between files. All samples of the same antibody were taken in the same microscopy session to allow comparison between files. To check for non-specific binding of the secondary antibody and to reject a false signal, the secondary antibody was applied to a separate set of samples under the same conditions, but without the primary antibody, and then also investigated in the same microscopy session.

## 3. Results

### 3.1. PC-3 RNA Expression Changes Under Simulated Microgravity

**Figure 2:**
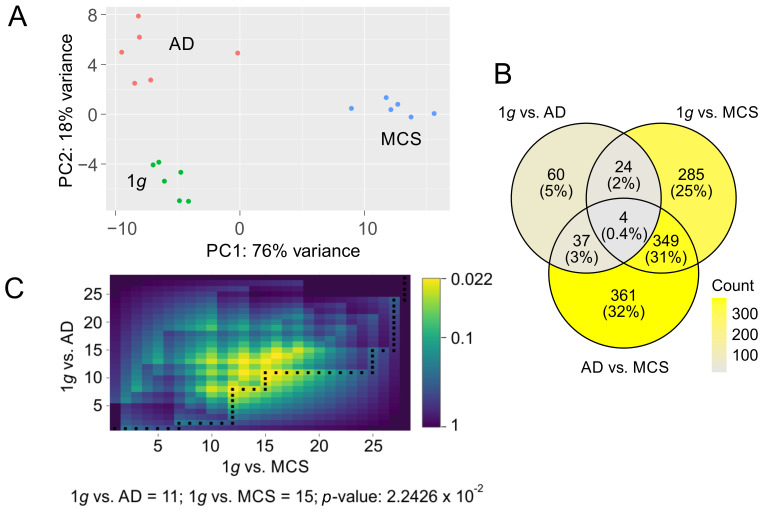
PCA of count data and representation of twofold deregulated genes with a false discovery rate (FDR) q-value of less than 10^−3^. (**A**) PCA of count data over the three conditions: 1*g* (green), AD (red), and MCSs (blue) (**B**) Distribution of DEGs over the three comparisons: 1*g* vs. AD, 1*g* vs. MCSs, and AD vs. MCSs. (**C**) DynaVenn determination of the most significant overlap between *p*-value-ranked 1*g* vs. AD DEGs and 1*g* vs. MCS DEGs.

The principal component analysis (PCA) of the count data from the 1*g* control (N = 6), adherent cells after three days on the RPM (AD, N = 6), and MCSs (N = 6) resulting from RPM rotation revealed a significant differentiation between the three conditions (Figure 2A). In particular, a clear discrimination of the MCS samples was observed with regard to the first principal component, which accounted for 76% of the dataset variance. Furthermore, a comparison of the three conditions revealed a larger variance of the AD samples with respect to the first two principal components (Figure 2A).

Considering the pronounced condition-dependent variation observed in PCA, and given that primary effects in a metabolic pathway cascade exert a pronounced influence on observed expression differences while simultaneously presumably offering the most accurate representation of them, we limited our analyses to genes exhibiting a highly significant differential expression. In total, we found 1120 genes to be twofold regulated (FDR < 10^−3^) in one or more of the three comparisons: control (1*g*, N = 6 samples) vs. adherent cells under s-µ*g* (AD, N = 6 samples); 1*g* vs. MCSs (N = 6 samples); AD vs. MCSs. Most differentially expressed genes (DEGs) were found in AD vs. MCSs (N = 751 genes) followed by the 1*g* vs. MCSs comparison (N = 662 genes) and 1*g* vs. AD (N = 125 genes, Table S1). Ratios of gene overlaps between the comparisons are documented in Figure 2B. We observed a significant ranking overlap of the most significant regulated genes in 1*g* vs. AD (first 11 genes) and 1*g* vs. MCSs (first 14 genes; *p* = 0.0224; Figure 2C).

Enrichment analyses demonstrate, on the one hand, the functional distribution of regulated genes and, on the other one, whether this assignment occurred significantly more frequently than in a random gene set. The multi-hierarchical structure of the GO annotation permits the multiple assignment of genes and the clustering of related annotations.

**Figure 3:**
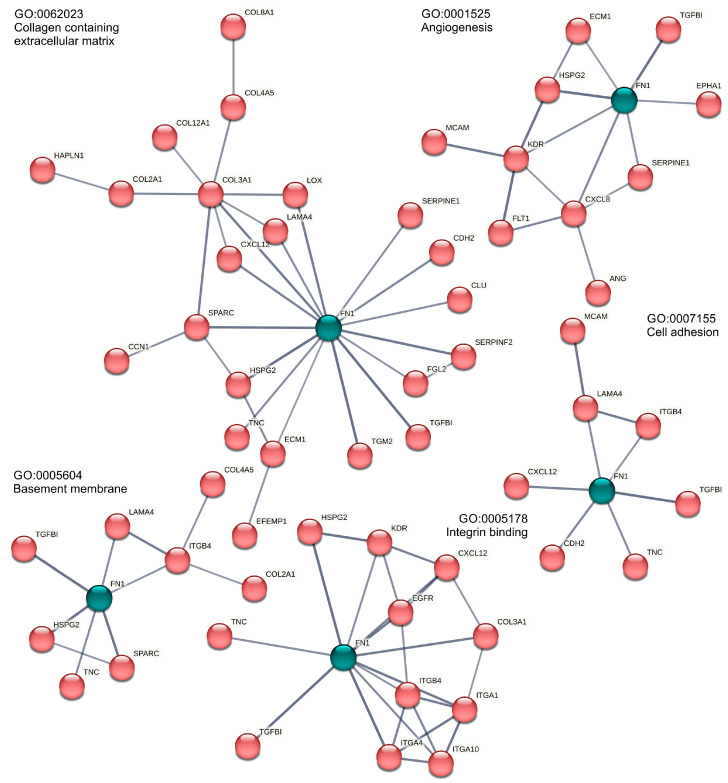
Enriched fibronectin (*FN1*, green)-related Gene Ontology annotations in the AD vs. MCSs comparison. Only highly confident (STRING score > 0.7) PPI connected genes are given. For better visibility, FN1 is highlighted in green against the red STRING networks.

In consequence, analogous enrichment clusters were discernible in the AD vs. MCSs comparison (Table 1) and the 1*g* vs. MCSs comparison (Table 2). On the one hand, the cell cycle and cell division kinetochore, spindle, and on the other, the cluster of extracellular space, angiogenesis, cell adhesion, and extracellular matrix organization are of particular interest with regard to cell–cell interaction. It is noteworthy that fibronectin frequently assumes a pivotal function in the annotation of this second category (Figure 3).

**Figure 4:**
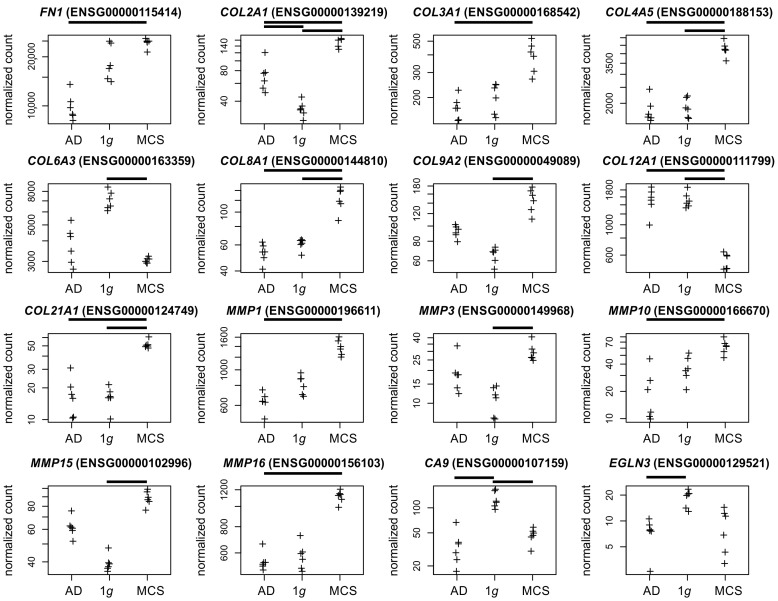
Normalized RNAseq read counts of sixteen candidate genes over the s-µ*g* conditions: AD (**left**), MCSs (**right**), and the 1*g* control samples (**center**). Given are fibronectin (*FN1*), differentially expressed collagen, and matrix metalloproteinase-coding genes and the hypoxia markers *CA9* and *EGLN3*. Bars on top of the graph indicate a twofold significant (padj < 10^−3^) differential expression between the conditions. Crosses represent single datapoints.

Collagen and matrix metalloproteinases. We found eight collagen genes regulated in the MCS formation (*COL2A1*, *COL3A1*, *COL4A5*, *COL8A1*, *COL12A1*, *COL21A1* in the AD to MCSs comparison; *COL2A1*, *COL4A5*, *COL8A1*, *COL12A1*, *COL21A1*, *COL6A3* and *COL9A2* in the 1*g* to MCSs comparison, Figure 4) which led together with the regulated matrix metalloproteases to the Gene Ontology GO:0062023 (collagen-containing extracellular matrix) annotation.

**Figure 5:**
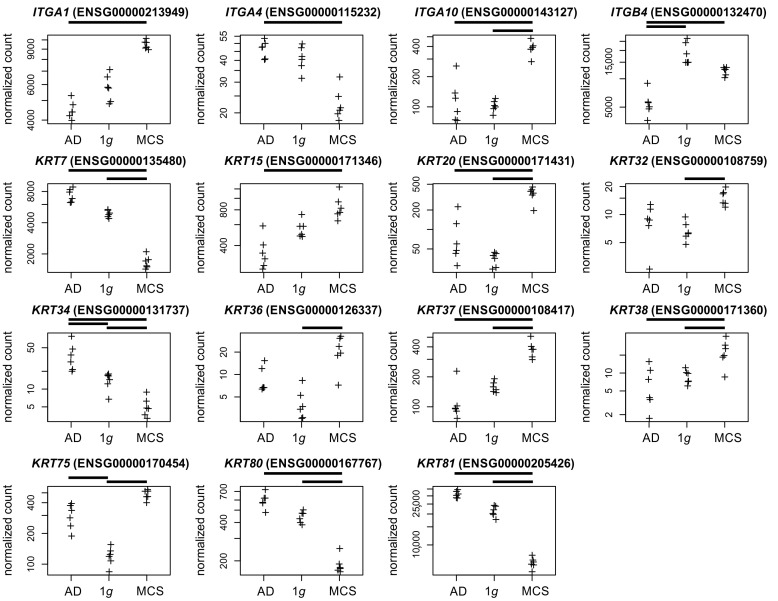
Normalized RNAseq read counts of fifteen candidate genes over the s-µ*g* conditions: AD (**left**), MCSs (**right**), and the 1*g* control samples (**center**). Given are differentially expressed integrins and keratin-coding genes. Bars on top of the graph indicate the twofold significant (Padj < 10^−3^) differential expression between the conditions. Crosses represent single datapoints.

Integrins. Integrins constitute a family of transmembrane proteins playing a role in cell–cell contact and cell–extracellular matrix (ECM) adhesion. A significant regulatory effect was observed between AD cells and MCSs regarding the integrins *ITGA1*, *ITGA4*, *ITGA10*, and *ITGB4* following RPM exposure (Figure 5).

Keratin. The keratin family of intermediate filament proteins is found in epithelial cells. These proteins span the cell from the nucleus to the cell junctions, performing a scaffold function that is crucial for maintaining cellular integrity and regulating internal cellular transport [36]. The keratin profile observed in the developing human prostate epithelium undergoes a transition from a relatively simple pattern to a more complex one [37]. In our study keratins, were regulated in 1*g* vs. AD (*KRT34/75*), 1*g* vs. MCSs (*KRT7/20/32/34/36/37/38/75/80*), and AD vs. MCSs (*KRT7/15/20/34/37/38/80/81*, Figure 5).

**Figure 6:**
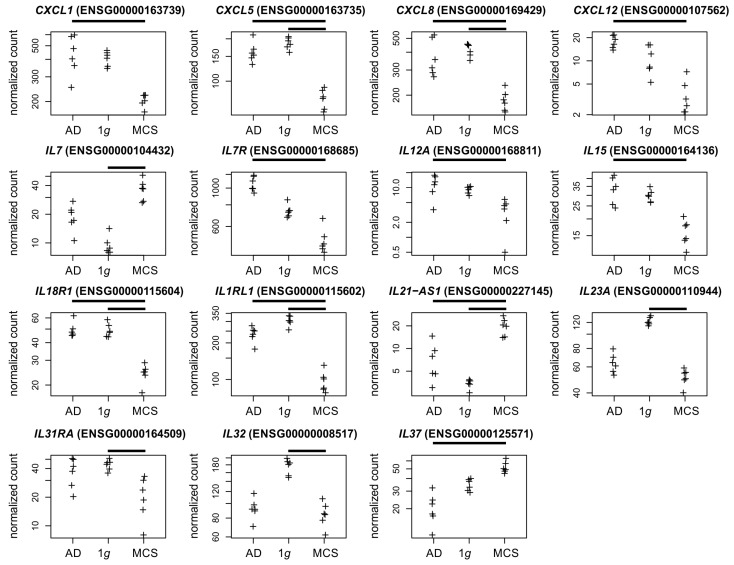
Normalized RNAseq read counts of fifteen cytokine-related genes over the s-µ*g* conditions: AD (**left**), MCSs (**right**), and the 1*g* control samples (**center**). Given are differentially expressed chemokine-coding genes, and interleukin or interleukin-related genes. Bars on top of the graph indicate a twofold significant (padj < 10^−3^) differential expression between the conditions. Crosses represent single datapoints.

Chemokines and interleukins. In a previous study, we demonstrated that the *CXCL1* chemokine, which is associated with the growth and progression of tumors, is upregulated in PC-3 cells subjected to parabolic flight campaign (PFC) gravitational stress [4]. It is noteworthy that under s-µ*g* conditions in the current study, *CXCL1* expression exhibited considerable heterogeneity in AD cells and was depleted in MCSs (Figure 6). This underscores the fact that gene regulation may occur in a manner that differs from that observed in the s-µ*g*-experiment and under short-term gravitational stress [7]. It should be noted that the four regulated chemokines were found to be depleted in MCSs (Figure 6).

**Figure 7:**
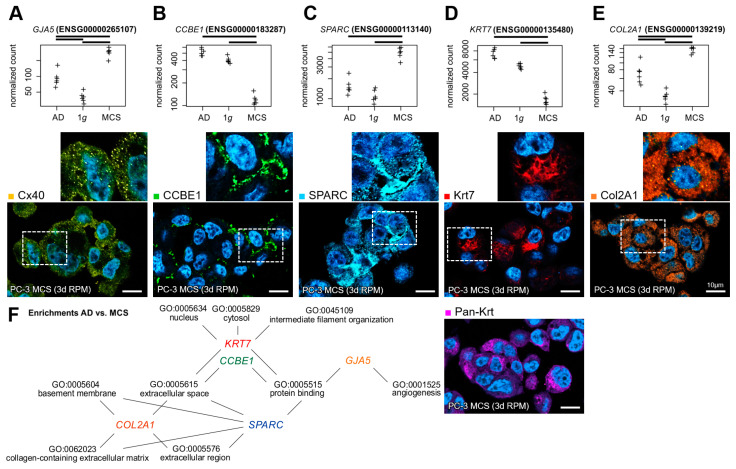
Gene and protein expression of important markers in desktop-RPM-generated PC-3 spheroids. Normalized RNAseq read counts (upper row) of five candidate genes and the cellular localization of their gene products (lower row) in 3-day-old MCSs: (**A**) connexin-40 (Cx40), (**B**) collagen and calcium-binding EGF domain-containing protein 1 (CCBE1), (**C**) osteonectin (SPARC), (**D**) keratin-7 (Krt7) and pan-keratin (pan-Krt), (**E**) collagen II α1 (Col2A1). The outlined area shows the magnification of the field of view (small images). Nuclei are indicated using DAPI staining (blue). Scale bars: 10 µm. (**F**) The five candidate genes were found to be involved in multiple significant GO enrichments of the AD to MCSs comparison DEGs. Crosses represent single datapoints.

Fluorescence staining of the gene products of five DEGs with high significance in the 1*g* to MCSs and AD to MCSs comparisons, in conjunction with multiple Gene Ontology (GO) enrichments (Figure 7F), was employed to visualize the cellular localization of the proteins within the MCSs. The protein products of the upregulated genes (*GJA5* coding for connexin-40 (Cx40), *SPARC* coding for osteonectin, *COL2A1* coding for collagen II α1) were similarly represented in all MCS cells (Figure 7A,C,E). The expression of the genes for calcium-binding EGF domain-containing protein 1 (CCBE1) and keratin-7 (Krt7) were downregulated on average in the MCSs. The respective proteins were only present in some spheroid cells: CCBE1 more in the marginal cells and Krt7 more in the central cells of the MCSs (Figure 7B,D).

### 3.2. Differential DNA Methylation Under Simulated Microgravity

**Figure 8:**
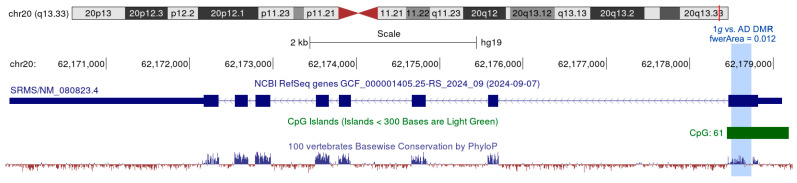
UCSC view of the chr20:62,170,900-62,179,500 region. The 1*g* vs. AD DMR is marked in blue.

At a significance level of 5% FDR, we found 11,090 differentially methylated positions (DMPs) and one differentially methylated region (DMR) in the 1*g* (N = 6 samples) to AD (N = 5 samples) comparison, but 10,797 DMPs and no DMRs in the 1*g* to MCSs (N = 5 samples) comparison (Table S3). The AD vs. MCSs comparison with five vs. five samples was underpowered and failed to yield significant DMPs or DMRs after correction for multiple testing. The significant 247 bp DMRs in the 1*g* vs. AD comparison (padj = 0.012) were located cytogenetically at 20q13.33 or genomically at chr20:62,178,498-62,178,744 (hg19) in the first exon of *SRMS* in a conserved region of a UCSC-annotated CpG island (Figure 8).

The gene-based enrichment analyses showed no significant result in either DMP dataset. The selected gometh CpG enrichment analysis takes into account the bias caused by the annotation of CpGs to multiple genes as well as the bias caused by the different number of CpGs annotated per gene.

**Figure 9:**
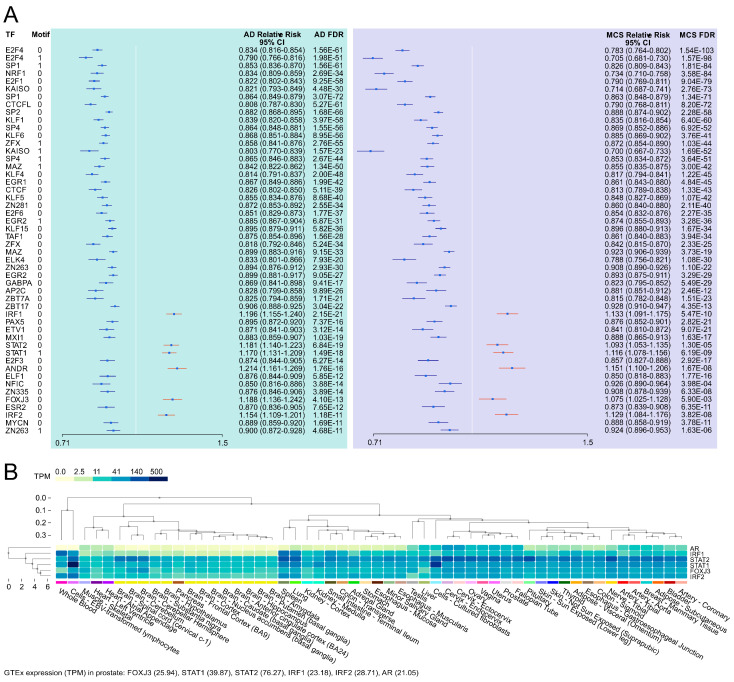
The HOCOMOCO and chi-square-based TFBS enrichment in µ*g*-induced differential CpG methylation loci. (**A**) Forest plot of TFBS enrichments (*p* < 10^−10^) near DMP loci and (**B**) GTEx multi-tissue expression of TF genes with positive enrichment in 1*g* vs. AD DMPs and 1*g* vs. MCS DMPs.

As illustrated in the forest plot in Figure 9, we identified 50 transcription factors (TFs) with transcription factor binding site (TFBS) enrichment in the vicinity of the 1*g* vs. AD or the 1*g* vs. MCSs differentially methylated positions (DMPs) at a TFBS analysis false discovery rate (FDR) of 10^−10^. Although the 45 negative TFBS enrichments are challenging to interpret from a biological standpoint, we identified positive enrichments of TFBS in the TF’s AR (androgen receptor); the interferon regulatory factors IRF1 and IRF2; and the TFs STAT1, STAT2, and FOXJ3 (Figure 9).

## 4. Discussion

The present study aimed to investigate the impact of s-µ*g* on transcriptional (RNAseq) and epigenetic DNA methylation (EPIC bisulfite array) in the neoplastic prostate cell line PC-3, which is a widely used model in PC research. In addition to the epigenetic results (Figure 8 and Figure 9), the following discussion addresses the significance of transcriptionally regulated collagens, matrix metalloproteinases (Figure 4), integrins, keratins (Figure 5), chemokines, and interleukins (Figure 6) in relation to microgravity and PC.

### 4.1. Transcriptional RPM Effects in PC-3 Cell Cultures

**Figure 10:**
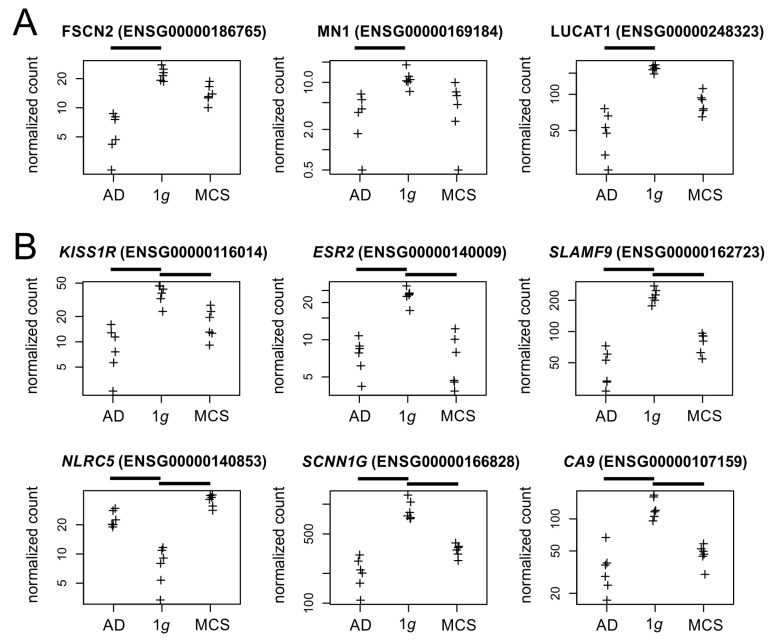
Normalized RNAseq read counts (**A**) of three candidate genes regulated between 2D cell culture under static (1*g*) and RPM conditions and (**B**) six genes regulated by the RPM exposure. Crosses represent single datapoints.

The culture effects of PC-3 cells under s-µ*g* are very complex. When specifically comparing a 2D cell culture under static (1*g*) and RPM conditions, 60 genes are significantly altered in their transcription after 3 days of culture (Supplementary Table S1). Among the most strongly regulated factors are genes for fascin (*FSCN2*), which cross-links actin into filament bundles, and some proto-oncogenes (*MN1*, *LUCAT1*, Figure 10A). Future research will show to what extent these expression changes lead to the often-observed morphological changes of 2D growing cells in µ*g*, such as the altered formation of actin stress fibers [38,39]. It would also be interesting to see to what extent the down-regulated pro-oncogenes are related to the presumed lower aggressiveness of cancer cells under µ*g* conditions [40].

Additionally, 24 other genes are significantly altered by RPM exposure regardless of the culture type of PC-3 cells (2D/3D) (Supplementary Table S1). These genes mainly code for surface receptors (*KISS1R*, *ESR2*, *SLAMF9*, *NLRC5*), ion channels (*SCNN1G*), and other transmembrane proteins (*CA9*, Figure 10B). *KISS1R* is the gene for a metastasis suppressor protein, whose downregulation in s-µ*g* may be involved in the observed in vitro metastasis of cancer cells on the RPM [6]. In contrast to PC-3 cells, an up-regulation of estrogen receptor transcription was observed in RPM cultures of breast and lung cancer cells [41,42] and led to estrogen-mediated stabilization of the formed tumor spheroids by Calu-3 cells.

### 4.2. Transcriptional Effects in RPM-Induced PC-3 Tumor Spheroids

With 361 significantly altered gene expressions, the transition from a 2D monolayer to a 3D spheroid culture of PC-3 cells on the RPM was accompanied by most transcriptional changes (Supplementary Table S1). Genes that were transcriptionally affected by the RPM included factors closely associated with growth behavior, such as ECM components, cell–ECM (integrins), and cell–cell junctions. In addition, the expression of communication factors such as chemokines/cytokines was also influenced by the RPM. The final enrichment analyses of the significantly differentially expressed genes revealed that the enriched GO categories were frequently matched in both the AD vs. MCSs and the 1*g* vs. MCSs comparisons. This is not unexpected given that 32% of the genes were matched (Figure 2B). The matches were primarily related to cell cycle-associated GO categories (see Table 1 and Table 2), but also encompassed the KEGG pathway “cell cycle” itself (hsa04110). Moreover, the GO categories “collagen-containing extracellular matrix” (GO:0062023), “angiogenesis” (GO:0001525), and “cell adhesion” (GO:0007155) were identified in both comparisons. The STRING network analysis of the AD vs. MCSs GO-annotated and differentially expressed genes revealed the presence of *FN1* at the center of those protein networks (Figure 3). A similar observation was made regarding the genes belonging to the GO category “integrin binding” (GO:0005178) in the AD vs. MCSs comparison (Figure 3). The enrichments associated with *FN1* are related to the gene groups of integrins and collagens. Collagens are in turn associated with metalloproteinases in the enriched GO category “extracellular matrix organization” (GO:0030198). Furthermore, the enriched GO annotation “intermediate filament organization” (GO:0045109) is mainly characterized by keratins. Fibronectin has been demonstrated to influence the metabolism of PC-3 cells as well as that of the androgen-sensitive prostate adenocarcinoma cell line LNCaP, which was established from a lymph node metastasis. While the LNCaP cell line shows relatively higher proliferation and apoptosis rates compared to the PC-3 cells, following propagation on fibronectin, cell proliferation and apoptosis were decreased among both cell lines [43].

Fibronectin is a key cellular adhesion molecule. As such, it has been shown to be involved in the process of 3D cellular aggregate formation. Caicedo-Carvajal et al. demonstrated that soluble fibronectin mediates tissue cohesion and a shift towards elastic behavior in 3D cellular aggregates via α5β1 integrin-fibronectin interactions [44]. Similarly, Robinson et al. reported that fibronectin can act as a substitute for external mechanical support to promote 3D cell aggregate compaction and cohesion, while inhibition of the fibronectin matrix assembly leads to cell dispersal [45]. Finally, a proteomic study on two thyroid cancer cell lines (FTC-133 and CGTH W-1) cultured in simulated µ*g* in an RPM for three days identified fibronectin as a key protein involved in spheroid formation [46].

Collagens. In two previous studies, we demonstrated the involvement of *COL1A1* in PC-3 MCS formation after a 24 h and 3-day RPM exposure and *COL4A5* after a 3-day RPM exposure, respectively [9,10]. Collagen, the major component of the stromal matrix, facilitates cancer progression and metastasis. Furthermore, the biomechanical signals induced by collagen alterations in the tumor microenvironment trigger a cascade of biological events [47]. For collagen, the most abundant molecule in the extracellular matrix (ECM), matrix metalloproteinases (MMPs), are the only enzymes capable of degradation. According to a study by Pal and coworkers [48], fibronectin treatment induces signaling involving FAK, PI-3K, Akt, and NF-κB, followed by upregulation of *MMP9* and *MMP1* in PC-3 cells. Interestingly, Singh et al. described that *CXCL12* increased *MMP2* expression in LNCaP and PC-3 cells [49]. We demonstrated in this study that the *CXCL12* (Figure 6) expression, but not the *MMP2* or *MMP9* expressions, are regulated in MCSs. The five regulated MMPs in PC-3 MCSs are also involved in the carcinogenesis of other types of cancer. *MMP1* and *MMP10* are expressed in oral squamous cell carcinoma (OSCC) tissues and are considered prognostic indicators [50,51]. Three of four hub genes identified as NF-κB-regulated in active ulcerative colitis [52] were also regulated in PC-3 MCS in our study (*CXCL1, MMP1*, and *MMP10*; Figure 4 and Figure 6).

Integrins. *ITGA1* has been linked to the progression of tumors and to a worse prognosis. *ITGA1* expression depletion resulted in the suppression of migration and invasion in hepatocellular carcinoma cells [53]. Furthermore, the *ITGA1* and *ITGA2* expression synergistically impacted PC prognosis and progression [54]. In human chondrocytes, an increase in the *ITGA10* expression was observed following exposure to gravitational stress during a parabolic flight comprising 31 parabolas. In addition, immunofluorescence microscopy revealed disruptions of the β-tubulin, vimentin, and cytokeratin networks [55]. The non-collagenous glycoprotein SPARC (Secreted Protein, Acidic, Cysteine-Rich alias Osteonectin, Figure 7C), a matricellular ECM protein that is nearly absent in normal mammary stroma but mediated by β4-integrin, is strongly expressed in breast cancer stroma [56,57,58]. Previous studies have shown a gravitational sensitivity of the *SPARC* gene expression and protein secretion. Human neural stem cells exhibit an increased SPARC secretion under real µ*g* [59]. Similarly, a twofold increase in *SPARC* gene expression was observed in mouse lung tissues after a 13-day space mission [60]. In contrast, a reduced SPARC expression was observed in the mouse osteoblast cell line 7F2 exposed to an RPM simulating Mars, moon, and microgravity [61]. SPARC is believed to be a tumor progression biomarker because of its expression in neoplastic cells of primary PC samples from metastatic cases [62]. In PC-3 cells with high tumorigenic and metastatic potential, SPARC was reported to be the major diffusible factor [63].

Chemokines and interleukins. The osteotropic factor *CXCL5* under 1*g* and the s-µ*g* condition itself is known to increase the syncytin-A expression in preosteoclast cells, which has an effect on osteoclast formation [64]. We suspect that *CXCL5* regulation could be related to the PC-3 origin from a bone metastasis. In an earlier study with a focus on the PC-3 cell line, we measured increased *CXCL8* gene expression in the MCS group compared to 1*g* of a 24 h RPM experiment [9]. The µ*g* effects on the inflammatory response of various cell types and multiple systems of the human body are significant. With respect to the inflammatory process in gastric mucosal epithelial cells, *CXCL8* was identified as one of 21 hub genes from a protein–protein interaction network (PPI) following culture in a rotary cell culture system (RCCS) bioreactor [65]. Interleukin-7 (IL-7), acting via the IL-7 receptor (IL-7R), plays a significant role in tumor progression, is closely associated with a poor prognosis for patients with PC, and is associated with MMP-3 and MMP-7 expression [66,67]. It is noteworthy that the current study has revealed a discrepancy between the observed upregulation of *IL7* and the depletion of *IL7R* in MCSs (Figure 6), which suggests the involvement of additional regulatory mechanisms. In the thyroid cancer cell line FTC-133, the *MMP3* and *IL15* gene expressions were similarly regulated under RPM and spaceflight conditions [7]. In human PC tissues, *IL12A* and *CD163* gene expressions were correlated and associated with a shorter survival [68]. The upregulation of *IL32* has been shown to act as a tumor suppressor in several cancers, including PC [69].

### 4.3. The RPM Cell Culture Reduces Hypoxia-Related Gene Expression in Bubble-Free Filled Culture Flasks

Reaction to hypoxia (GO:0001666) was the only enriched GO term when comparing 2D cultures in bubble-free cell culture flasks under 1*g* and RPM conditions. That rotation of these cell cultures counteracts hypoxia, which is caused by the reduced gas exchange surface, has already been shown in previous RPM studies [70]. In the RPM, rotation creates fluid flows that enable better mixing of the medium [71].

Hypoxia is associated with increased metastasis, prognosis, radio resistance, and poor survival in PC patients [72]. The expression levels of *CA9* and *EGLN2* are significantly upregulated under hypoxic conditions in the malignant melanoma cell line A375 [73]. In contrast, we observed a depletion of *CA9* and *EGLN3* (Egl-9 Family Hypoxia Inducible Factor 3) RNA expression in PC-3 cells under s-µ*g* conditions (Figure 4), which suggests the presence of a hypoxia reaction that may be absent or insufficient.

### 4.4. DNA Methylation Changes Under Simulated Microgravity

DMPs and the DMR. A total of 21,887 significant DMPs at a significance level of 5% FDR is in notable contrast to the single DMR observed in this study. It seems that a comparison of six 1*g* samples with five RPM-exposed samples (in AD or MCS) is sufficient for the detection of DMPs but suboptimal for the detection of DMRs. Overall, 20% of the 125 1*g* vs. AD DEGs and 24.6% of the 662 1*g* vs. MCS DEGs could be assigned to one or more DMPs. The significant DMR in the 1*g* to AD comparison is located in the first exon of the gene Src-related kinase lacking C-terminal regulatory tyrosine and N-terminal myristoylation sites (*SRMS*). The tyrosine kinase *SRMS*, first described in 1994 [74], has not been reported in PC or in the context of µ*g* experiments. In platinum treatment of ovarian cancer, ROS-activated SRMS deceases MKK4-JNK activation thereby contributing to resistance to platinum-based chemotherapy [75]. *SRMS* overexpression in colorectal cancer is associated with a poor prognosis. In silico analyses revealed evidence of cytokine–cytokine receptor interaction and chemokine involvement in *SRMS* contribution to colorectal cancer development [76].

TFBS enrichments. The enrichment analysis across the two DMP datasets yielded no significant findings. In contrast, we identified positive transcription factor binding site (TFBS) enrichments for five transcription factors in the vicinity of the DMPs: AR, IRF1, IRF2, STAT1, STAT2, FOXJ3. Genes of all five TFs were expressed in GTEx [77] prostate samples (Figure 9B). The androgen receptor (AR) gene expression is downregulated under µ*g* in rat testis [78,79]. AR plays a pivotal role in prostate organogenesis, is known to be critical for the initiation and progression of PC [80], and is a key therapeutic target for hormone-naïve-advanced PC and castration-resistant PC [81]. Furthermore, AR variations play a role in therapeutic resistance [82]. Cheng and colleagues discovered in 2020 that the transcription factor Interferon Regulatory Factor 1 (IRF1) acts as a tumor suppressor in an AR-mediated transcriptional regulatory network in PC [83]. Moreover, using comprehensive mass spectrometric analyses the interferon regulatory factor 1 (IRF1) has been identified as a key transcription factor in growth arrest of an LNCaP-based mTOR overexpressing PC cell line [84]. The micro-RNA miR-221 is significantly upregulated under the gravitational stress of parabolic flight conditions [4], directly inhibits the expression of *SOCS3* and *IRF2*, and regulates the STAT1/STAT3-mediated activation of the JAK/STAT signaling pathway in PC [85]. The combination of AR and JAK2/STAT1 inhibition resulted in a significant reduction in tumor growth, which was attributed to the diminished capacity for DNA damage repair and the increased propensity for apoptosis [86]. The sixfold dihydrogenphosphate ester of inositol, inositol hexakisphosphate (IP6), causes reduced caspase-3 activation, reduced DNA fragmentation, and upregulation of NF-κB-responsive (IκB-alpha, IRF-2) genes in PC-3 cells [87]. FOXJ3 plays a role in cell proliferation, migration, and invasion in lung cancer by MiR-517a-3p regulation [88]. In colorectal cancer, miR-27a and FOXJ3 dysregulate the mitochondrial dynamics [89]. In breast cancer, FOXJ3 regulation of snail expression has an influence on cell proliferation and migration [90].

## 5. Conclusions

The aim of our study was to identify transcriptional and epigenetic responses to s-µ*g* exposure and MCS cell aggregation, to classify them in silico using enrichment and PPI analyses, and finally to derive gravisensitive PC candidate genes and gene interactions. RPM exposure of PC-3 cells induced changes in their morphology (Figure 1), and 3D aggregates were detected. The gene expression of cytoskeletal genes, genes of the extracellular matrix, growth factors, cytokines, and focal adhesion genes, among others, were significantly altered. These data agree with earlier results about changes in the gene and protein expression obtained for several cell types [5,33,36,64]. Moreover, a significant upregulation of genes belonging to the PAM pathway, together with activation of VEGF and MAPK signaling, indicated their involvement in the cellular changes occurring in µ*g* [4,5]. Furthermore, the differential expression of cytokines like IL-1α, IL-1β, IL-6, and IL-8 impacts metastatic growth of PC cells [4]. The PC relevance of the candidates was discussed based on literature references in order to identify potential PC biomarkers. In addition to fibronectin, these could be identified among the collagens, matrix metalloproteins, integrins, keratins, chemokines, and interleukins. We are aware that these potential PC biomarkers based on only one cell line need to be validated in further studies, but our study provides the basis to limit these validations to a few promising genes in a cost-effective way. Changes in gene expression in response to a stimulus (e.g., s-µ*g*) or because of cell aggregation (e.g., MCSs) and their effect on carcinogenesis form a tight network and could partly confirm and complement previous results. The regulation of *FN1* in the AD vs. MCSs gene expression comparison apparently shows a central role in some GO annotations associated with extracellular matrix formation in the PPI view (Figure 3) but must be relativized due to the relatively high and thus MCS-like *FN1* expression under 1*g* conditions. Nevertheless, due to the relevance of the networks to carcinogenesis and the relationship of some of the contributing genes to µ*g* effects, a coherent picture of the MCS gene network emerges, complemented by the epigenetic and TFBS findings.

## Figures and Tables

**Table 1:** David enrichment (<5% FDR) of the annotations KEGG, GO cellular component (CC), GO biological process (BP), and GO molecular function (MF) over 751 genes which were twofold differentially expressed between the two s-µ*g* conditions of AD and MCSs (details in Table S2).

Comparison	Term	Count	FDR
AD vs. MCSs	KEGG hsa04110—cell cycle	26	1.87 × 10^−8^
	GO BP GO:0051301—cell division	36	5.20 × 10^−6^
	GO CC GO:0005615—extracellular space	102	1.05 × 10^−5^
	GO CC GO:0005576—extracellular region	104	1.05 × 10^−5^
	GO CC GO:0005654—nucleoplasm	165	1.38 × 10^−4^
	GO CC GO:0062023—collagen-containing extracellular matrix	31	3.51 × 10^−4^
	GO BP GO:0000278—mitotic cell cycle	19	3.54 × 10^−4^
	GO BP GO:0001525—angiogenesis	25	3.89 × 10^−4^
	GO MF GO:0005515—protein binding	437	7.54 × 10^−4^
	GO BP GO:0007156—homophilic cell adhesion via plasma membrane adhesion molecules	20	0.0013
	GO CC GO:0005694—chromosome	22	0.0015
	GO BP GO:0007155—cell adhesion	35	0.0037
	GO BP GO:0007059—chromosome segregation	14	0.0041
	GO CC GO:0000775—chromosome, centromeric region	10	0.0047
	GO CC GO:0000776—kinetochore	16	0.0065
	GO CC GO:0005604—basement membrane	12	0.0065
	GO CC GO:0005819—spindle	15	0.0075
	GO BP GO:0006268—DNA unwinding involved in DNA replication	7	0.0083
	GO CC GO:0005829—cytosol	203	0.0083
	GO CC GO:0071162—CMG complex	5	0.0083
	GO MF GO:0005178—integrin binding	17	0.0128
	GO CC GO:0005634—nucleus	217	0.0146
	GO BP GO:0000070—mitotic sister chromatid segregation	8	0.0169
	GO BP GO:0045109—intermediate filament organization	11	0.0169
	GO BP GO:0030335—positive regulation of cell migration	22	0.0248
	GO CC GO:0032133—chromosome passenger complex	4	0.0282
	GO MF GO:0005524—ATP binding	73	0.0292
	GO MF GO:0004714—transmembrane receptor protein tyrosine kinase activity	8	0.0292
	GO BP GO:0030198—extracellular matrix organization	16	0.0365
	GO CC GO:0045121—membrane raft	16	0.0420

**Table 2:** David enrichment (<5% FDR) of the annotations KEGG, GO cellular component, GO biological process, and GO molecular function over genes which were twofold differentially expressed (0.1% FDR) between 1*g* and the *s-*µ*g* conditions of MCSs (N = 662) and AD (N = 125), respectively (details in Table S2).

Comparison	Term	Count	FDR
1*g* vs. MCSs	GO BP GO:0051301—cell division	37	3.46 × 10^−8^
	GO BP GO:0007059—chromosome segregation	18	1.17 × 10^−6^
	GO CC GO:0000776—kinetochore	21	3.17 × 10^−6^
	GO BP GO:0000278—mitotic cell cycle	19	3.15 × 10^−5^
	GO CC GO:0005819—spindle	18	6.38 × 10^−5^
	GO BP GO:0007156—homophilic cell adhesion via plasma membrane adhesion molecules	20	1.79 × 10^−4^
	GO BP GO:0045109—intermediate filament organization	13	1.84 × 10^−4^
	GO CC GO:0005576—extracellular region	88	2.17 × 10^−4^
	GO MF GO:0008017—microtubule binding	25	2.45 × 10^−4^
	GO BP GO:0007094—mitotic spindle assembly checkpoint signaling	9	3.19 × 10^−4^
	GO BP GO:0000070—mitotic sister chromatid segregation	9	7.47 × 10^−4^
	KEGG hsa04110—cell cycle	18	7.58 × 10^−4^
	GO CC GO:0005615—extracellular space	81	0.0029
	GO BP GO:0090307—mitotic spindle assembly	9	0.0079
	GO CC GO:0062023—collagen-containing extracellular matrix	25	0.0080
	GO CC GO:0000940—outer kinetochore	5	0.0080
	GO BP GO:0001525—angiogenesis	20	0.0087
	GO CC GO:0015630—microtubule cytoskeleton	16	0.0110
	GO BP GO:0007052—mitotic spindle organization	10	0.0126
	GO BP GO:0007155—cell adhesion	30	0.0126
	GO MF GO:0005524—ATP binding	67	0.0143
	GO MF GO:0030280—structural constituent of skin epidermis	8	0.0143
	GO MF GO:0030020—extracellular matrix structural constituent conferring tensile strength	9	0.0143
	GO CC GO:0000152—nuclear ubiquitin ligase complex	4	0.0148
	GO CC GO:0030496—midbody	15	0.0148
	GO CC GO:0072686—mitotic spindle	13	0.0148
	GO CC GO:0031012—extracellular matrix	17	0.0148
	GO BP GO:0000281—mitotic cytokinesis	10	0.0155
	GO CC GO:0043231—intracellular membrane-bounded organelle	41	0.0161
	GO CC GO:0009986—cell surface	32	0.0189
	GO BP GO:0051983—regulation of chromosome segregation	5	0.0235
	GO BP GO:0030198—extracellular matrix organization	15	0.0239
	GO CC GO:0005581—collagen trimer	10	0.0278
	GO CC GO:0005829—cytosol	174	0.0284
	GO CC GO:0005886—plasma membrane	172	0.0311
	GO CC GO:0005874—microtubule	19	0.0383
	GO MF GO:0005515—protein binding	371	0.0391
1*g* vs. AD	GO BP GO:0001666—response to hypoxia	8	0.0150

## Data Availability

Data are contained within the article and Supplementary Materials and raw and processed data will be provided on request.

## Supplementary Materials

The following supporting information can be downloaded at: https://www.mdpi.com/article/10.3390/biom15020303/s1, Table S1: Supplementary Data S1; Table S2: Supplementary Data S2; Table S3: Supplementary Data S3.

## Abbreviations

The following abbreviations are used in this manuscript:

1*g*	1*g* control
AA	African American (population)
AD	adherent cells after three days in the RPM
ChIP-Seq	chromatin immunoprecipitation DNA sequencing
DAVID	Database for Annotation, Visualization and Integrated Discovery
DEG	differentially expressed gene
DMP	differentially methylated position
DMR	differentially methylated region
EA	European American (population)
ECACC	European Collection of Authenticated Cell Cultures
ECM	extracellular matrix
FCS	fetal calf serum
FDR	false discovery rate (*p*-value multiple testing adjusted by the Benjamini–Hochberg method)
TFBS	transcription factor binding site
JEB-PA	junctional epidermolysis bullosa with pyloric atresia
MCS	multicellular spheroid
MSC	mesenchymal stem cell
NSCLC	blood of non-small cell lung cancer
OSCC	oral squamous cell carcinoma
PA-JEB	pyloric atresia-junctional epidermolysis bullosa syndrome
PC	prostate cancer
PF	parabolic flight
PPI	protein-to-protein interaction
PTC	premature termination codon
padj	multiple testing adjusted *p*-value
RPM	random positioning machine
cf-mtDNA	cell-free mitochondrial DNA
cfDNA	cell-free DNA
iRPM	incubator random positioning machine
s-µ*g*	simulated microgravity
